# The Journal of the American Medical Association

**Published:** 1889-01

**Authors:** 


### THE JOURNAL OF THE AMERICAN MEDICAL ASSOCIATION.

Dr. N. S. Davis, who has been the editor of the *Journal of the American Medical Association* since its foundation in 1883, resigned the editorship some months ago, his resignation taking effect on January 1. The Board of Trustees of the *Journal* elected Dr. John B. Hamilton, Surgeon-General of the U. S. Marine Hospital Service, as editor, and he has arrived in Chicago to take charge of the *Journal*, having been granted leave of absence by the Treasury Department.

There is an erroneous impression that the office of publication will be removed from Chicago; it will remain in this city.

